# The impact of adverse childhood experiences on mental health, sexual risk behaviors, and alcohol consumption in adulthood

**DOI:** 10.3389/fpsyt.2024.1352824

**Published:** 2024-04-10

**Authors:** Miguel Landa-Blanco, Gabriela Vásquez, Gretel Portillo, Federico Sproviero, Yarani Echenique

**Affiliations:** School of Psychological Sciences, National Autonomous University of Honduras, Tegucigalpa, Honduras

**Keywords:** adverse childhood experiences, flourishing, anxiety, depression, somatization, selfreported health, sexual risk behavior, alcohol consumption

## Abstract

The purpose of the study was to determine how Adverse Childhood Experiences (ACE) relate to adulthood flourishing, symptoms of depression, anxiety, somatization, self-reported health, sexual risk behaviors, and alcohol consumption. A quantitative cross-sectional methodology was used. A total of 452 adults completed the survey. The most prevalent ACE include physical abuse (44.69%), separation/divorce of parents (41.81%), living with someone with alcohol problems (39.38%), and being sworn, insulted, or humiliated by adults at home (35.62%). Almost one out of every four respondents (24.34%) reported being touched by an adult, 17.92% reported that an adult tried to manipulate the respondent into touching them, and 8.19% were forced to have sexual intercourse. Results indicate that women reported a higher number of ACE than men. The number of ACE is inversely related to flourishing and self-reported health; while being positively associated with participant’s scores in depression, anxiety, somatization, sexual risk behaviors, and alcohol use. The regression model, including the eleven ACE and respondents’ sex and age, achieved medium effect sizes for somatization, depression, and anxiety symptoms and small effect sizes for flourishing, self-reported health, sexual risk behaviors, and alcohol consumption. Specific ACE have a particularly significant negative impact on mental health outcomes: forced intercourse, witnessing familial violence, verbal humiliation, and living with individuals struggling with mental health issues and drug consumption or who were incarcerated. In conclusion, the study highlights the alarming prevalence of ACE among the Honduran population and their significant negative impact on mental health outcomes during adulthood.

## 1 Introduction

Early life experiences are vital in shaping the brain, emotions, social skills, and physical health. These experiences can profoundly impact a person’s overall health and well-being in adulthood (1). The World Health Organization (WHO) estimates that one in every eight people worldwide has some mental disorder (2). Several factors contribute to the prevention or development of mental health disorders. Emotional skills developed in the family, quality education, and constructive human relationships are critical protective factors. On the other hand, vulnerability factors include extreme poverty, experiences of violence, physical punishment, and harsh upbringing, among others, which are considered adverse (3).

Childhood experiences, those occurring before the age of 18, can be categorized as either benevolent or adverse. Benevolent Childhood Experiences (BCE) encompass a positive self-concept, the presence of at least one secure caregiver, supportive teachers, good neighbors, and more. These circumstances are conducive to improved mental health outcomes and the cultivation of positive, functional social, and familial connections (4, 5). In contrast, Adverse Childhood Experiences (ACE), including neglect, physical and psychological abuse, parental separation, incarceration, or household substance abuse, are considered harmful (5). Such maltreatment often leads to a range of issues in children, from interpersonal difficulties to academic struggles (6).

Early exposure to stress can significantly impact cognitive, emotional, and social development, even contributing to premature death (7). Coping mechanisms adopted by those with ACE experiences tend to be avoidant and emotion-focused, often resulting in poorer mental health outcomes later in life (8), including anxiety, depression, and suicidal thoughts (9). Moreover, ACE can lead to psychological trauma, triggering emotional responses such as anguish, panic attacks, and feelings of worthlessness, which can hinder overall development and disrupt social relationships (10, 11). While ACE have been associated with various outcomes in adulthood, this study will focus on its relationship to flourishing, depression, anxiety, somatization, self-reported health, sexual risk behaviors, and alcohol consumption. A concise overview of each variable follows in the subsequent paragraphs.

Flourishing refers to positive human aspects related to general well-being, including self-esteem, a sensation of purpose in life, optimism, supportive social relationships, and “good” feelings (12). ACEs are expected to have a negative impact on adult flourishing. The accumulation of ACEs can lead to increased stress, impaired cognitive development, and disrupted emotional regulation, all of which can hinder an individual’s ability to achieve flourishing in adulthood (13). Moreover, ACE can erode social support networks and limit opportunities for personal growth and development (14, 15), further impeding flourishing outcomes.

On the other hand, depression is characterized by extreme and persistent feelings of sadness that impact the person in several aspects of their life; it includes physical symptoms, loss of interest or pleasure in doing things, excessive guilt, and suicidal thoughts (16). ACE, encompassing various forms of maltreatment, neglect, and household dysfunction, have been consistently linked to an increased risk of depression in adulthood (17, 18). Depression symptoms can stem from a lack of secure attachment with caregivers during childhood. This can lead to neurobiological changes that affect an individual’s ability to form relationships, maintain a sense of personal identity, and regulate emotions (19). Additional evidence also suggests that people who reported ACE experienced a prolonged response to stress stimuli, making them more prone to developing depressive symptoms (20).

Anxiety is characterized by intrusive and agitated thoughts, with a tense sensation that can also have physical symptoms like high blood pressure, trembling, and palpitations (16). The inclusion of adult anxiety as an outcome stems from research suggesting a significant correlation between traumatic childhood events and increased vulnerability to anxiety disorders later in life (21, 22).

Somatization describes when a psychological disturbance, like emotional and psychological distress, manifests in physical symptoms (16), like aches and pains, weakness, or shortness of breath that interferes with the person’s daily living (23). Previous studies have found a link between ACE and somatization in adulthood (24). Such a relationship persists even after controlling for age, sex, and formal education. The dynamic between ACE and adulthood somatization is particularly evident for specific events, such as emotional and sexual abuse (25). The appearance of psychosomatic symptoms in adulthood is closely related to psychological trauma in the person’s life. For example, survivors of childhood sexual abuse (CSA) present a high incidence of somatic symptoms that do not have any pre-established medical explanation (26). Findings suggest that CSA induces short-term biological changes that may contribute to the development of psychiatric and medical illnesses in adulthood. Victims of CSA also have a greater incidence of engaging in behavior risks (27), lower positive mental health (28), deteriorated health-related quality of life (29), conversion disorder, borderline personality disorder, anxiety, depression, post-traumatic stress disorder, schizophrenia, and substance abuse (30).

Self-reported health is a subjective rating of how a person evaluates his/her health (31). The relationship between ACE and health outcomes during adulthood has been well-documented in the literature (1). For instance, people reporting ACE are at higher risk of smoking excessively, diabetes, arthritis, cardiovascular problems, and Sexually Transmitted Diseases (STDs) (32). Sexual risk behaviors refer to actions or practices that increase the likelihood of negative sexual health outcomes, such as the transmission of sexually transmitted infections or unintended pregnancies. These behaviors may include an early sexual debut, higher number of sexual partners, having sexual intercourse under the influence of alcohol or drugs, unprotected sex, sexual compulsivity, and others (33, 34).

Alcohol consumption refers to the voluntary intake of alcoholic beverages. Problematic alcohol consumption refers to excessive or harmful patterns of drinking that lead to adverse physical, social, or psychological consequences (35). In general terms, the cumulative number of reported ACE is positively related to an increase consumption of alcohol during adulthood (36). Previous studies also suggest that people who, as children, experienced household abuse were more likely to engage in alcohol consumption behaviors, including binge drinking (37).

To understand the dynamic between ACE and mental health, contextual factors must be considered. In this sense, Honduras is one of the most violent countries in the world, with children and adolescents constituting a high proportion of the country’s homicide victims (38). Adverse experiences in childhood occur frequently (39), with many practices, like corporal punishment and family violence, being a common occurrence in the Honduran population. This is of particular concern, considering that children depend on their families to survive and meet their essential affective needs (40).

Honduras is also considered one of the poorest countries in the region (41). This poverty level is important because it means people cannot afford to receive mental health assistance in a private facility as they don’t have the means to pay for it. According to the report of the Pan American Health Organization (PAHO) the burden of disorders mental disorders in the country has its highest prevalence in behavioral disorders during adolescence (42). Then, in early adulthood, anxiety and depression-related disorders are highly prevalent; common, but to a lesser extent, are self-harm disorders, somatoform disorders, substance and/or alcohol use disorders, eating disorders, schizophrenia, and bipolarity, among others (42). This growing concern is becoming a common problem in public health, as the demand for professional mental health services is high, but the country’s response capabilities are limited. Honduras only has 0.66 psychiatrists and 0.62 psychologists per 100.000 inhabitants, unlike countries with a higher human development index that report 43.96 psychiatrists and 84.14 psychologists per 100.000 inhabitants (43). The country’s number of pediatric psychiatrist is yet lower, with only 0.01 per 100.000 inhabitants (44).

The country’s public health system is not only grappling with a shortage of trained mental health professionals, but also contending with widespread stigmas associated with mental health (45). Individuals who have received a psychiatric diagnosis may encounter discrimination from their social environment, leading to a reluctance to seek assistance owing to apprehension of being stigmatized as “mentally unstable” or “hazardous” (43).

Adverse Childhood Experiences (ACE) and their contributing factors have been profoundly studied in developed countries (46, 47). However, research on the topic is still scarce in the Honduran setting. So, while results from the international context are the foundation for new research, they might not be accurate for the Honduran population as the difference between culture, beliefs, access to resources, and others cannot be unseen. Therefore, it is imperative to study these factors and their impact on mental health during adulthood in the country so that culturally appropriate interventions might be developed and applied to the Honduran people, aimed to improve the psychological impact of these ACE’s.

Consequently, the purpose of the study was to determine how Adverse Childhood Experiences relate to adulthood flourishing, symptoms of depression, anxiety, somatization, self-reported health, sexual risk behaviors, and alcohol consumption, as stated in the following hypotheses:

- Hypothesis 1: The number of reported ACE will correlate negatively with flourishing and positively with depression, anxiety, somatization, self-reported health, sexual risk behaviors, and alcohol consumption.
- Hypothesis 2: Respondents who reported specific ACE will score lower on flourishing and higher on depression, anxiety, somatization, self-reported health, sexual risk behaviors, and alcohol consumption.

## 2 Materials and methods

### 2.1 Participants

A total of 452 participants completed the survey, including 300 (66.37%) women and 152 (33.63%) men. The respondents’ ages varied between 18 and 68 years, averaging 29.96 (*SD* ± 8.98). Participants were recruited through a non-probabilistic sampling, specifically through volunteers and snowball methods. The survey was posted in several social media groups and academic emails. As inclusion criteria, participants were required to be Hondurans 18 years or older at the time of the data collection.

### 2.2 Instruments

#### 2.2.1 Adverse childhood experiences

The Spanish version of the Behavioral Risk Factor Surveillance System (BFRSS), Module 21, of Adverse Childhood Experiences was used. Eleven categorical questions regarding specific adverse events experienced before the respondent turned 18 are included (48): ACE-1: living with someone depressed/mentally ill/suicidal; ACE-2: living with someone with problematic alcohol consumption; ACE-3: living with someone with drug consumption problems; ACE-4: living with someone who was incarcerated; ACE-5: parental separation/divorce; ACE-6: domestic violence; ACE-7: childhood physical abuse; ACE-8: verbal insults/humiliations; ACE-9: being sexually touched by a person five years older than the respondent; ACE-10: manipulated into sexually touching a person five years older than the respondent; ACE-11: forced sexual intercourse. A dicotomical yes (1)/no (0) response system was used in the present research.

#### 2.2.2 Flourishing scale

The Spanish version of the Flourishing Scale (FS) is an 8-item Likert-type scale with a seven-point response system (1= “totally disagree”; 7= “totally agree”); higher summative scores indicate a higher self-reported flourishing (49). The psychometric properties of the FS (Spanish version) have been previously established as valid and reliable measurements for the Honduran population (50). Based on data from the current study, the FS achieved an adequate internal consistency with a McDonald’s *Ω* of 0.90, *CI* 95% [.88,.91], and a unidimensional structure based on EFA fit indices (RMSEA=0.07, SRMR=.03, TLI=0.97, CFI=0.98).

#### 2.2.3 Patient health questionnaire-9

Symptoms of depression were assessed using the Patient Health Questionnaire-9 (PHQ-9); this is a nine-item Likert-type scale in which respondents report the frequency of symptoms experienced during the two weeks before the assessment (51). Each symptom is evaluated on a four-point response system (0= “not at all”; 3=“ nearly every day”); higher summative scores indicate a higher depressive symptomatology. Based on data from the current study, the PHQ-9 achieved adequate internal reliability with a McDonald’s *Ω* of 0.91, *CI* 95% [.90;.92], and a unidimensional structure based on EFA fit indices (RMSEA=.07, SRMR=0.03, TLI=0.96, CFI=0.97).

#### 2.2.4 Generalized anxiety disorder-7

Symptoms of anxiety were assessed using the Generalized Anxiety Disorder-7 (GAD-7); this is a seven-item Likert-type scale in which respondents report the frequency of symptoms experienced during the two weeks before the assessment (52). Each symptom is evaluated on a four-point response system (0= “not at all”; 3= “nearly every day”); higher summative scores indicate higher anxiety symptomatology. Given the current data, the GAD-7 has an adequate internal consistency with a McDonald’s *Ω* of 0.91, *CI* 95% [.92;.94], and a unidimensional structure based on EFA fit indices (RMSEA=0.07, SRMR=.02, TLI=0.98; CFI=0.98).

#### 2.2.5 Somatic symptom scale-8

The Somatic Symptom Scale –8 (SSS-8) is an eight-item Likert-type scale in which respondents report the frequency of somatization symptoms experienced during the two weeks before the assessment. Each symptom is evaluated on a four-point response system (0= “not at all”; 3=“ nearly every day”); higher summative scores indicate higher somatization (23). In our study, the SSS-8 achieved an adequate internal consistency with a McDonald’s *Ω* of 0.88, *CI* 95% [.86,.89], and a unidimensional factor structure based on EFA fit indices (RMSEA=0.09, SRMR=.04, TLI=0.94, CFI=0.95).

#### 2.2.6 Self-reported health item

The Self-reported Health Item (SRH), contains a single question: “in general, how would you rate your health” (31). Response options use a 4-point Likert type set (1= “good”, 4= “very poor”. However, in our study, the SRH was inversely recoded post-data collection so that higher scores indicate better self-reported health. Previous validations of the SRH suggest adequate predictive validity regarding short-term mortality (<10 years) (31).

#### 2.2.7 The sexual risk behaviors scale

The Sexual Risk Behaviors Scale (SRBS) is a 5-item Likert-type scale with a 5-point response set (1= “never”; 5= “very often”). Higher summative scores indicate a higher prevalence of sexual risk behaviors (34). Based on data from the current study, the SRBS has an adequate internal consistency, with a McDonald’s Omega of 0.74, *CI* 95% [0.70; 0.78], and a unidimensional factor structure based on results from EFA (RMSEA=0.09, SRMR=0.04, TLI=0.91, CFI=0.96).

#### 2.2.8 Alcohol use disorder identification test for consumption-abbreviated

The Alcohol Use Disorder Identification Test for Consumption-Abbreviated (AUDIT-C) measures alcohol consumption in adults using three items, each one with a different response set, although every question uses a 4-point scale (53). Higher summative scores indicate a higher consumption of alcohol. Based on data from the current study, the AUDIT-C is considered a reliable measurement, with a McDonald’s *Ω* of 0.88, *CI* 95% [0.86; 0.90], and a unidimensional factor structure based on results from EFA (RMSEA=0.01, SRMR=0.01, TLI=0.99, CFI=0.99).

### 2.3 Ethical considerations

Informed consent was required from all participants. The data was collected anonymously. At the end of the survey, respondents were presented with various website links containing psychological resources and platforms that provide free psychological attention. The study was approved by the Research Ethics Committee of the Faculty of Social Sciences (National Autonomous University of Honduras), under registration CEIFCS-2023-P1.

### 2.4 Data analyses

Summative scores were determined for each scale. Then, using JASP (54), Mcdonald’s Omega (*Ω*) was used to estimate internal consistency; 95% Confidence Intervals (*CI*) were calculated. An Exploratory Factor Analysis (EFA) was run to assess the factor structure of each scale. Fit indices were reported for EFA; these include: Root Mean Square Error of Approximation (RMSEA), Standardized Root Mean Square Residual (SRMR), Tucker Lewis Index (TLI), and Comparative Fit Index (CFI). Mean (M) scores and standard deviations (SD) were calculated for the summative score of each scale. Additionally, the prevalence of each ACE was assessed through absolute and relative frequencies. All hypotheses were tested at a 95% confidence level. A chi-square test (*χ*²) was used to determine if the presence/absence of each ACE was associated with respondents’ sex; contingency coefficients (*CC*) were calculated as effect size estimates of these associations.

Bivariate correlations were calculated using Pearson’s *r* coefficient. A multiple linear regression model was independently run for every outcome (summative scores). The input included the 11 individual ACE as predictors (1=yes; 0=no), respondent’s sex and age; no further data was manipulated beyond determining raw summative scores. Each model reports the adjusted determination coefficient (*r* ^2^) and effect size (*f* ^2^). A post-hoc power analysis was made for each regression using G*Power (55), specified as a fixed model *r* ^2^ deviation from zero. The input parameters included the effect size (*f* ^2^), probability of committing a type I error (*α=*0.05), total sample size (*n*=452), and number of predictors in the model (including the 11 ACE, respondents’ sex, and age).

## 3 Results

Of the total sample, 17.48% (*n*=79) reported zero ACE, 19.03% (*n*=86) reported one ACE, 29.87% (*n*=135) reported between two and three ACE and 33.62% (*n*=152) reported more than 4 ACE, see **Table 1**.

**Table 1:** Frequency analysis of the number of reported ACE.

Number of ACE	Counts	% of Total	Cumulative %
0	79	17.48	17.48
1	86	19.03	36.51
2 to 3	135	29.87	66.38
4 or more	152	33.62	100

The total sample size was 452 adults.

On average, women (*M*=2.99; *SD* ± 2.34) reported a significantly higher number of ACE than men (*M*=2.35; *SD* ± 2.29), *t*=-2.76, *p*<.001, *d*=-0.27. The most prevalent ACE include physical abuse (ACE-7) (44.69%), separation/divorce of parents (ACE-6) (41.81%), living with someone with alcohol problems (ACE-2) (39.38%), and being sworn, insulted or humiliated by adults at home (ACE-8) (35.62%). Almost one out of every four respondents (24.34%) reported being touched by someone older (ACE-9), 17.92% reported that someone older tried to manipulate the respondent into touching them (ACE-10), and 8.19% were forced to have sexual relationships (ACE-11). ACE-8, ACE-9, and ACE-10 are significantly associated with the respondents’ sex (*p*<.05), with a higher proportion of women experiencing those specific ACE; see **Table 2**.

**Table 2:** Frequency analysis of adverse childhood experiences.

ACE	Total *n* (%)		Men *n* (%)		Women *n* (%)		Chi-square test
	No	Yes	No	Yes	No	Yes	
ACE1. Did you live with anyone who was depressed, mentally ill, or suicidal?	336 (74.34%)	116 (25.66%)	119 (78.29%)	33 (21.71%)	217 (72.33%)	83 (27.67%)	*χ*²=1.88; *p*=.17; *CC*=0.06
ACE2. Did you live with anyone who was a problem drinker or alcoholic?	274 (60.62%)	178 (39.38%)	95 (62.50%)	57 (37.50%)	179 (59.67%)	121 (40.33%)	*χ*²=0.34; *p*=.56; *CC*=0.03
ACE3. Did you live with anyone who used illegal street drugs or abused prescription medications?	392 (86.73%)	60 (13.27%)	130 (85.53%)	22 (14.47%)	262 (87.33%)	38 (12.67%)	*χ*²=0.29; *p*=.59; *CC*=0.03
ACE4. Did you live with anyone who served time or was sentenced to serve time in a prison, jail, or other correctional facility?	410 (90.71%)	42 (9.29%)	136 (89.47%)	16 (10.53%)	264 (91.33%)	26 (8.67%)	*χ*²=0.41; *p*=0.52; *CC*=0.03
ACE5. Were your parents separated or divorced?	263 (58.19%)	189 (41.81%)	97 (63.82%)	55 (36.18%)	166 (55.33%)	134 (44.67%)	*χ*²=2.98; *p*=0.08; *CC*=0.08
ACE6. Did you live with parents or adults who slapped, hit, kicked, punched, or beat each other up?	375 (82.96%)	77 (17.04%)	129 (84.87%)	23 (15.13%)	246 (82.00%)	54 (18.00%)	*χ*²=0.59; *p*=0.44; *CC*=0.04
ACE7. Not including spanking, (before age 18), did a parent or adult in your home ever hit, beat, kick, or physically hurt you in any way?	250 (55.31%)	202 (44.69%)	87 (57.24%)	65 (42.76%)	163 (54.33%)	137 (45.67%)	*χ*²=0.34; *p*=0.56; *CC*=0.03
ACE8. Did a parent or adult in your home ever swear at you, insult you, or put you down?	291 (64.38%)	161 (35.62%)	108 (71.05%)	44 (28.95%)	183 (61.00%)	117 (39.00%)	*χ*²=4.45, *p*=.03; *CC*=0.10
ACE9. Did anyone at least five years older than you, or an adult, ever touch you sexually?	342 (75.66%)	110 (24.34%)	135 (88.82%)	17 (11.18%)	207 (69.00%)	93 (31.00%)	*χ*²=21.51, *p*<.001; *CC*=0.21
ACE10. Did anyone at least five years older than you or an adult, try to make you touch them sexually?	371 (82.08%)	81 (17.92%)	136 (89.47%)	16 (10.53%)	235 (78.33%)	65 (21.67%)	*χ*²=8.51; *p*<.001; *CC*=0.14
ACE11. Did anyone at least five years older than you or an adult, force you to have sex?	415 (91.81%)	37 (8.19%)	143 (94.08%)	9 (5.92%)	272 (90.67%)	28 (9.33%)	*χ*²=1.56; *p*=.21; *CC*=0.06

Significant associations are presented in bold. χ²=chi-square test, CC, Contingency Coefficient.

Bivariate correlation analysis shows that the reported ACE number is significantly and inversely related to flourishing and self-reported health. On the other hand, the number of reported ACE is positively related to participant’s scores in depression, anxiety, somatization, sexual risk behaviors, and alcohol use. Although secondary to the current study, other noteworthy correlations are evident. For instance, higher flourishing scores and self-reported health correlate significantly with low scores in depression, anxiety, and somatization. Sexual risk behaviors are positively associated with the number of reported ACE, depression, anxiety, somatization, and -more strongly- alcohol use. Altogether, these findings provide supporting evidence in favor of Hypothesis 1, “The number of reported ACE will correlate negatively with flourishing and positively with depression, anxiety, somatization, self-reported health, sexual risk behaviors, and alcohol consumption”, see **Table 3**.

**Table 3:** Bivariate correlations.

Variable	Statistic	1	2	3	4	5	6	7	8	*M* (*SD*)
1. Adverse childhood experiences	*r*	—								2.77 (2.34)
	*p*-value	—								
2. Flourishing	*r*	-0.15 **	—							45.71 (8.66)
	*p*-value	<0.01	—							
3. Depression	*r*	0.38 ***	-0.39 ***	—						10.65 (7.32)
	*p*-value	<.001	<.001	—						
4. Anxiety	*r*	0.29 ***	-0.30 ***	0.83 ***	—					8.27 (6.29)
	*p*-value	<.001	<.001	<.001	—					
5. Somatization	*r*	0.38 ***	-0.24 ***	0.75 ***	0.73 ***	—				10.19 (7.62)
	*p*-value	<.001	<.001	<.001	<.001	—				
6. Self-reported health	*r*	-0.14 **	0.32 ***	-0.29 ***	-0.26 ***	-0.33 ***	—			3.27 (0.65)
	*p*-value	<0.01	<.001	<.001	<.001	<.001	—			
7. Sexual risk behaviors	*r*	0.21 ***	-0.05	0.18 ***	0.18 ***	0.17 ***	-0.03	—		8.07 (3.49)
	*p*-value	<.001	.29	<.001	<.001	<.001	.51	—		
8. Alcohol consumption	*r*	0.11 *	-0.07	0.16 ***	0.14 **	0.10	-0.03	0.49 ***	—	2.42 (2.61)
	*p*-value	.02	.14	<.001	<.01	.06	.54	<.001	—	

Pearson’s r coefficient was used to determine the correlation coefficient between variables. Self-reported health was inversely coded in this table; higher scores indicate better self-reported health.

*p <.05, **p <.01, ***p <.001.

Based on their overall *F*-test, all outcomes were significantly explained by the regression model. However, a higher determination coefficient (*r^2^*) was achieved for the following outcomes: somatization (*r^2^* =.24), depression (*r^2^* =.23), and anxiety symptoms (*r^2^* =.21). Based on *f* ^2^, these coefficients achieve a medium effect size (56), see **Table 4**.

**Table 4:** Linear regression models for specific outcomes.

Predictor	Flourishing		Depression		Anxiety		Somatization		Health		Sexual risk behaviors		Alcohol Consumption	
	*β*	*p*	*β*	*p*	*β*	*p*	*β*	*p*	*β*	*p*	*β*	*p*	*β*	*p*
Intercept	41.66	<.001	14.98	<.001	11.63	<.001	10.96	<.001	3.12	<.001	7.87	<.001	2.97	<.001
ACE-1	-2.47	<0.01	2.97	<.001	2.12	<.001	2.55	<.001	-0.29	<.001	0.52	0.17	0.35	0.24
ACE-2	0.80	0.38	-1.11	0.11	0.70	0.25	-1.11	0.13	0.04	0.51	0.16	0.66	0.03	0.91
ACE-3	-0.57	0.66	1.52	0.13	1.77	0.04	3.50	<.001	-0.03	0.80	0.16	0.75	0.22	0.58
ACE-4	1.78	0.22	0.12	0.91	-0.80	0.41	0.88	0.44	0.13	0.25	1.31	0.02	0.28	0.53
ACE-5	0.15	0.86	-0.29	0.66	-0.81	0.15	-0.66	0.32	0.04	0.53	0.05	0.88	0.42	0.11
ACE-6	-1.88	0.12	2.03	0.03	2.28	<0.01	1.47	0.13	-0.01	0.99	0.89	0.07	0.25	0.50
ACE-7	-0.51	0.58	0.39	0.59	-0.07	0.91	0.87	0.24	-0.06	0.37	0.28	0.45	0.25	0.39
ACE-8	-0.84	0.41	1.50	0.05	1.57	0.02	1.11	0.16	-0.08	0.30	-0.11	0.79	-0.25	0.42
ACE-9	2.41	0.08	0.53	0.61	0.53	0.57	1.56	0.15	0.04	0.70	0.94	0.08	0.26	0.54
ACE-10	-1.35	0.36	0.87	0.44	0.28	0.78	1.52	0.20	-0.03	0.82	-0.93	0.12	-0.39	0.39
ACE-11	-4.24	<0.01	3.22	<0.01	1.68	0.14	1.91	0.16	-0.19	0.43	1.88	<.01	0.53	0.31
Sex	0.56	0.52	0.88	0.19	1.34	0.02	2.86	<.001	-0.11	0.08	-1.64	<.001	-0.99	<.001
Age	0.16	<.001	-0.23	<.001	-0.19	<.001	-0.17	<.001	0.01	<0.01	0.02	0.362	-0.01	0.43
*r* ^2^ adjusted	0.07		0.23		0.21		0.24		0.07		0.11		0.03	
F	3.56		11.43		10.10		12.03		3.52		5.09		2.01	
*f* ^2^	0.08		0.30		0.27		0.32		0.08		0.12		0.03	
Power	>0.99		>0.99		>0.99		>0.99		>0.99		>0.99		>0.99	

β represents unstandardized coefficients. ACE-1: living with someone depressed/mentally ill/suicidal; ACE-2: living with someone with problematic alcohol consumption; ACE-3: living with someone with drug consumption problems; ACE-4: living with someone who was incarcerated; ACE-5: parental separation/divorce; ACE-6: witnessing domestic violence; ACE-7: childhood physical abuse; ACE-8: verbal insults/humiliations; ACE-9: being sexually touched by a person five years older than the respondent; ACE-10: manipulated into sexually touching a person five years older than the respondent; ACE-11: forced sexual intercourse. Statistically significant differences are presented in bold. f^2^ is classified as follows: small (0.02), medium (.15) and large (.35) (56). Power for all regression models exceeded.99.

Somatization was positively related to living with someone who consumed drugs (ACE-3), living with someone depressed/mentally ill/suicidal (ACE-1) and being a woman; age has inverse effects on somatization. ACE-1 and ACE-2 positively explained depression symptoms, while age had inverse effects on the outcome. Anxiety symptoms were positively explained by ACE-1, ACE-2, ACE-3, ACE-6, ACE-8, and being a woman; age had an inverse effect on anxiety scores.

Sexual risk behaviors were positively predicted by having lived with someone who served time (ACE-4), being forced to have sex (ACE-11), and being male. Self-reported health is inversely related to living with someone depressed/mentally ill/suicidal (ACE-1) and being a woman, while age is positively related to better scores on this variable. Having been forced into having sex (ACE-11) and living with someone depressed/mentally ill/suicidal (ACE-1) significantly predicted lower flourishing scores, while age was positively related to higher flourishing outcomes. Alcohol consumption was not predicted significantly by any of the included ACE; however, being a woman is a protective factor. Altogether, these findings provide partial support for Hypothesis 2 “Respondents who reported specific ACE will score lower on flourishing and higher on depression, anxiety, somatization, self-reported health, sexual risk behaviors, and alcohol consumption”.

## 4 Discussion

Our findings suggest that ACE are highly prevalent among the Honduran sample. The most prevalent ACE included physical abuse, parental separation/divorce, living with someone with alcohol problems, and emotional abuse. The findings underscore a significant association between ACE and various mental health indicators, with reported ACE inversely related to flourishing and self-reported health and positively associated with depression, anxiety, somatization, sexual risk behaviors, and alcohol use. Specific ACE have a particularly significant negative impact on mental health outcomes for the Honduran population; these experiences include forced intercourse, witnessing familial violence, verbal humiliation, and living with individuals struggling with mental health issues and drug consumption or who were incarcerated. The regression model provides a comprehensive understanding of how ACE collectively contributes to mental health outcomes, with somatization, depression, and anxiety symptoms strongly influenced by ACE. Overall, the results indicate that experiencing adverse childhood events impacts the general well-being in adulthood, reducing positive aspects and increasing negative ones.

Thus, the findings emphasize the critical importance of incorporating ACE into mental health interventions, highlighting the necessity for customized approaches that specifically address individuals with distinct ACE backgrounds. This has significant practical implications for preventive public policy, as previous studies indicate a potential intergenerational connection between parental early maladaptive schemas and the emergence of similar schemas in their offspring. Recent insights from a systematic review revealed that parents’ disconnection and rejection schemas negatively influence the parent-child bond, impeding effective parenting and elevating the risk of schema transfer (57).

The identified associations between specific ACE and mental health outcomes in clinical settings provide valuable insights for healthcare professionals. Clinicians should be vigilant in screening for a history of ACE, particularly among women, as it may inform treatment strategies and interventions. Integrating trauma-informed care practices into healthcare settings can contribute to a more holistic and effective approach to managing mental health conditions associated with ACE. Additionally, pediatric healthcare settings play a vital role in the early identification, intervention, and treatment of adverse childhood experiences. Healthcare providers in pediatrics bear a crucial responsibility in screening for ACE and implementing customized interventions. With their continuous interactions among providers, children, and families, primary care settings foster trust and facilitate effective interventions, making them especially effective for ACE screening (58).

Other studies suggest that people with a greater number of ACE were more likely to seek help, both professionally and informally. The primary reasons for seeking help were anxiety, stress, and depression, with a higher prevalence of depression noted among individuals with more ACE. Additionally, those with more ACE reported more unmet needs and were more inclined to obtain health information from sources like schools, other professionals, or the media (59). This evidences the dire need for universally available interventions directed at people suffering from ACE.

At the therapeutic level, this study reinforces the importance of a trauma-informed approach, which understands that symptoms are a consequence of behavior resulting from poorly processed experiences from the past that have an impact on the present moment. Thus, understanding that anxiety and depression are only the tip of the iceberg and being able to provide patients with evidence-based treatment forms an integrative view of the person, which is often overlooked when only treating a symptom and not its root problem.

In this sense, from a psychological perspective, a recent systematic review suggests the high effectiveness of cognitive-behavioral interventions for treating mental health outcomes in people who reported ACE, with a particular focus on fostering resilience in the target population. Other interventions also show potentially promising results; these include psychoeducation, parental training, cross-sector support systems, and school-based intervention (60).

Moreover, interesting results emerge when comparing the prevalence of ACE between Hondurans and other countries. For instance, compared to the findings of the Behavioral Risk Factor Surveillance System ACE Data (USA) (61), the proportion of Hondurans reporting zero ACE stands at 17.48%, half the percentage of Americans at 36.1%. Hondurans reporting four or more ACE accounts for 33.62% of the sample, twice as much as the percentage reported in the USA. A more detailed examination reveals that instances of physical abuse reported by Hondurans are twice as prevalent (44.69%) as those reported by Americans (22.7%). In the Honduran cohort, the rate of parental divorce notably exceeds that of the USA, with 41.81% compared to 28.4%. Furthermore, a larger percentage of Hondurans (25.66%) indicated having cohabited with someone experiencing mental illness, in contrast to Americans (19.9%). It is worth noting that the prevalence of forced intercourse for the Honduran sample is similar to the one reported in a meta-analysis of worldwide studies on the topic (62).

These findings suggest several possible explanations. To illustrate, in 2017, Honduras allocated a mere 1.6% of its total health budget to mental health services (44), whereas, on average, high-income countries allocate 5% of their health budget to this sector (63). Socioeconomic disparities, including poverty (39), limited access to education (64), and healthcare, may heighten Hondurans’ vulnerability to ACE compared to other nations. The increased prevalence of physical abuse antecedents could stem from cultural norms that normalize certain disciplinary or violent practices within Honduran families (40). Moreover, the elevated rate of parental divorce among Hondurans, relative to Americans, may reflect cultural differences regarding attitudes towards marriage, alongside family dynamics and socioeconomic influences (65). Similarly, the higher proportion of Hondurans who cohabited with individuals experiencing mental illness could also be attributed to the prevalence of mental health problems within the country (44), coupled with cultural expectations regarding family obligations and caregiving responsibilities.

A more detailed perspective on the topic can be gained when considering the results from a recent study by Médecins Sans Frontières, which analyzed the structural factors impeding mental health in Honduras (44). Firstly, it highlights the pervasive impact of violence, encompassing homicides, domestic abuse, and sexual violence. Secondly, it underscores the vulnerability of specific groups, such as pregnant women, single mothers, unemployed individuals, children, those living in extreme poverty, victims of forced migration, and members of the LGBTQ+ community. Additionally, in line with prior research (66), the study highlights the repercussions of the COVID-19 pandemic on the well-being of the Honduran population. This emphasizes the need for improved access to mental health services in the country, as stigmas, limited response capabilities, the scarcity of psychologists and psychiatrists, and the lack of specialized pediatric institutions with a focus on mental health exacerbate this issue (43, 44, 67).

Our findings provide useful information for policymakers and practitioners in tailoring interventions that address the unique socio-cultural context of Honduras. Local stakeholders may include the country’s Ministries of Education, Health, and universities. For instance, the Ministry of Education must implement programs that address affected children’s psychosocial needs, integrate trauma-informed practices into school curricula, and provide educator training. Simultaneously, the Ministry of Health must enhance mental health services, ensuring accessibility and quality care for individuals impacted by ACE, through resource expansion, professional training, and support network establishment. In collaboration with the public health system, local universities can play a crucial role by organizing extension activities to identify and prevent adverse childhood experiences within specific contexts. Specialized pediatric mental health units, such as the Psychological Attention Center (CAPS) of the National Autonomous University of Honduras, may significantly contribute to the mental health of the ACE-affected population. Additionally, universities can conduct research to deepen understanding of childhood trauma prevalence and impact, informing evidence-based interventions and policy recommendations. This coordinated effort among governmental bodies and academic institutions is essential for addressing the complex challenges posed by childhood trauma in Honduras.

Despite the relevance of our study, important limitations must be considered when interpreting the research findings. For example, the cross-sectional design hampers the establishment of causal relationships, and reliance on self-reported measures introduces concerns about bias and recall errors. The limited sample size and exclusion of certain ACE constrain the generalizability of findings; thus, the results may not represent the Honduran population. Moreover, the absence of control for confounding factors, limited exploration of mechanisms, and a focus on ACE in isolation hinder a deeper understanding of their complex interplay with adulthood outcomes. The study fails to adequately address cultural and contextual considerations and resilience factors, underscoring the need for more nuanced and comprehensive research. Acknowledging these limitations, including the cross-sectional design and reliance on self-reported measures, advocates for ongoing nuanced and comprehensive research to deepen understanding of the intricate interplay between ACE and adulthood outcomes.

Future research should consider longitudinal designs to unveil the long-term trajectories of individuals with diverse ACE exposures, exploring resilience factors and coping mechanisms to inform targeted interventions. There is also a profound need to investigate the prevalence of ACE in certain key groups within the Honduran population, such as people living in extreme poverty, victims of forced migration, and members of the LGBTQ+ community (44, 68). Additional research could test the hypothesis that ACE mediates the relationship between socioeconomic conditions and mental health outcomes. Comparative studies across different nations could yield a valuable perspective into how cultural factors influence the connection between ACE and mental health in low-and middle-income countries. Finally, incorporating qualitative approaches could capture lived experiences, provide subjective insights, and refine intervention strategies for a more comprehensive understanding of the implications of ACE on adult mental health.

In conclusion, our study highlights the pervasive prevalence of Adverse Childhood Experiences (ACE) in Honduras and their profound impact on mental health outcomes. Specific ACE, such as physical abuse and parental divorce, exhibit significant associations with depression, anxiety, and other adverse mental health indicators. These findings underscore the critical importance of integrating ACE into mental health interventions and policy frameworks tailored to the unique socio-cultural context of Honduras. Collaboration among stakeholders, including healthcare professionals, policymakers, and academic institutions, is essential to implement targeted interventions that mitigate the long-term consequences of childhood trauma and promote resilience among the population. Ongoing research efforts, incorporating longitudinal designs and qualitative approaches, are imperative to deepen understanding of the complex interplay between ACE and adulthood outcomes, informing evidence-based interventions and policy recommendations.

## Data availability statement

The raw data supporting the conclusions of this article will be made available by the authors, without undue reservation.

## Ethics statement

The studies involving humans were approved by Comité de Ética en Investigación de la Facultad de Ciencias Sociales (UNAH). The studies were conducted in accordance with the local legislation and institutional requirements. The participants provided their written informed consent to participate in this study.

## Author contributions

ML: Writing – review & editing, Writing – original draft, Supervision, Project administration, Methodology, Investigation, Formal Analysis, Conceptualization. GV: Writing – review & editing, Writing – original draft, Investigation, Conceptualization. GP: Writing – review & editing, Writing – original draft, Investigation, Conceptualization. FS: Writing – review & editing, Writing – original draft, Investigation, Conceptualization. YE: Writing – review & editing, Writing – original draft, Investigation, Conceptualization.
